# Cardioprotective Potential of Polyphenolic Rich Green Combination in Catecholamine Induced Myocardial Necrosis in Rabbits

**DOI:** 10.1155/2015/734903

**Published:** 2015-08-26

**Authors:** Fatiqa Zafar, Nazish Jahan, Khalil-Ur-Rahman, Ahrar Khan, Waseem Akram

**Affiliations:** ^1^Department of Chemistry, University of Agriculture, Faisalabad 38000, Pakistan; ^2^Department of Biochemistry, University of Agriculture, Faisalabad 38000, Pakistan; ^3^Department of Pathology, University of Agriculture, Faisalabad 38000, Pakistan; ^4^Department of Entomology, University of Agriculture, Faisalabad 38000, Pakistan

## Abstract

The present study was designed to develop safer, effective, and viable cardioprotective herbal combination to control oxidative stress related cardiac ailments as new alternatives to synthetic drugs. The synergetic cardioprotective potential of herbal combination of four plants *T. arjuna* (T.A.), *P. nigrum* (P.N), *C. grandiflorus* (C), and *C. oxyacantha* (Cr) was assessed through curative and preventive mode of treatment. In preventive mode of treatment, the cardiac injury was induced with synthetic catecholamine (salbutamol) to pretreated rabbits with the proposed herbal combination for three weeks. In curative mode of treatment, cardiotoxicity/oxidative stress was induced in rabbits with salbutamol prior to treating them with plant mixture. Cardiac marker enzymes, lipids profile, and antioxidant enzymes as biomarker of cardiotoxicity were determined in experimental animals. Rabbits administrated with mere salbutamol showed a significant increase in cardiac marker enzymes and lipid profile and decrease in antioxidant enzymes as compared to normal control indicating cardiotoxicity and myocardial cell necrosis. However, pre- and postadministration of plant mixture appreciably restored the levels of all biomarkers. Histopathological examination confirmed that the said combination was safer cardioprotective product.

## 1. Introduction

Cardiovascular diseases have become a global threat to life [1] and are major reason of 17.1 million fatalities every year. It is expected that death toll due to cardiac diseases will reach up to 20 million in 2020 [2]. In Pakistan, the condition has become really alarming as cardiac ailments contribute to about 25% of deaths in the country [3]. Diverging to the consistent efforts of medical and pharmaceutical scientists to combat the heart diseases, rather than to minimize the prevalence, the numbers of cardiac patients are increasing [4]. Currently available synthetic cardioprotective medicines have not only been related to a number of side effects but are also very costly [5]. The easy availability, comparatively less side effects, and low cost of medicinal plants make them more attractive therapeutic agents [6].

Medicinal plants enriched with polyphenols, possessing free radical scavenging potential, may reduce the risk of heart diseases because of inverse relationship between cardiovascular diseases and intake of polyphenols [7]. Free radicals are reactive species generated in the body as a result of many endogenous (metabolic pathways) and exogenous (environmental pollution, pesticides, and exposure to radiations) sources [8]. Different environmental factors elevate the level of free radicals and cells become unable to work efficiently against the free radicals leading to accumulation of radicals and oxidative stress which is involved in cell damage, necrosis, and apoptosis and has main causative role in pathogenesis of cardiovascular diseases [9, 10]. Many antioxidants like Vitamins C and E and plant polyphenols are efficient tools in oxidative stress and cardiovascular disorders as potential therapeutic agents [11].

Various medicinal plants possess certain preventive effects regarding heart diseases [12]. Botanical therapeutics with multicomponent has several advantages over single plant extract/isolated compound that may earn them a more prominent place in the field of herbal medicines. Multicomponent therapeutics offer bright prospects for the control of many diseases in a synergistic manner [13].

Mixtures of interacting bioactive compounds produced by plants may provide important combination therapies that simultaneously affect multiple pharmacological targets and provide clinical efficacy beyond the reach of single compound-based drugs. Therefore four medicinal plants were selected to evaluate their combined cardioprotective potential. Medicinal plants* Crataegus oxyacantha *(Cr) exhibit hypotensive, cardiotonic, antispasmodic, diuretic, and sedative properties. It helps to treat heart disease by dilating peripheral and coronary blood vessels and improves the supply of blood to the heart and extenuating symptoms in early period of heart failure [14].* Cactus grandiflorus* (C) is particularly useful in treating different ailments associated with the heart and is a very good source of polyphenols. It has the ability to reduce the oxidative stress due to its powerful antioxidant activity [15].* Piper nigrum* (P.N) commonly known as Black Pepper is used to treat cardiac diseases, being a very good combination of antioxidants.* Terminalia arjuna* (T.A) has significant antioxidant properties and is a good heart tonic [16]. Gemmomodified extract of this plant (T.A (g)) is a rich source of bioactive substances. Gemmo preparations (freshly growing parts) of medicinal plants are important as these contain many active substances that start to disappear as plant reaches maturity [17].

Finding ways to screen the synergistic combinations from numerous herbal pharmacological agents is still an ongoing challenge. In the present research work extracts of the above four medicinal plants being used by alternative practitioners and those have known folk medicinal background were used in the ratio of (C : Cr : P.N : T.A (g) = 2 : 1 : 2 : 2) for the assessment of synergetic cardioprotective activity. These plants have been previously analyzed by our research group for their individual antioxidant potential. In the present research, synergistic cardioprotective potential of the combination was evaluated in salbutamol induced cardiotoxicity through animal model.

## 2. Methodology

### 2.1. Sample Collection

Freshly growing leaves (gemmo parts) of medicinal plant* Terminalia arjuna* (Arjun) were collected from the Botanical garden, University of Agriculture, Faisalabad, and got identified from plant taxonomist at the Department of Botany, University of Agriculture, Faisalabad, Pakistan.* Piper nigrum* (Black pepper) was bought from market and ground into fine powder. Ethanolic extracts of medicinal plants* Cactus grandiflorus* and* Crataegus* were purchased from a branded company of Germany “Schwabe” from Homoeopathic Medical store.

### 2.2. Sample Preparation

Freshly growing leaves (gemmo parts) of* Terminalia arjuna* were washed with cold water to remove dirt and were used in the form of gemmomodified extract.* Piper nigrum* was purchased from herbal store and was ground into fine powder, whereas prepared ethanolic extracts of Cactus and Crataegus were used.

### 2.3. Preparation of Plant Extracts

Gemmomodified extract of* Terminalia arjuna* was prepared by maceration process. The fresh plant material was blended in a mixture of alcohol and glycerin having 2 : 1 ratio for 21 days [17]. Aqueous extract of* Piper nigrum* was prepared by boiling the plant material with water for ten minutes and filtrate was used.

### 2.4. Determination of Phenolics by HPLC

For the determination of phenolic contents by HPLC, method of Pak-Dek et al. [18] was followed. Plant extract (50 mg) was dissolved in 24 mL methanol and homogenized, and then distilled water (16 mL) and HCl (10 mL, 6 M) were added. This mixture was thermostated for 2 h at 95°C. The final solution was filtered using a 0.45 *µ*m nylon membrane filter and High Performance Liquid Chromatography (HPLC) analysis was carried out. The conditions used for the HPLC analysis are given in Table 1.

### 2.5. Preparation of Herbal Combinations

Herbal combination was prepared by appropriately mixing the extracts of Cactus, Crataegus, Arjuna, and* Piper nigrum* in the ratio of 2 : 1 : 2 : 2. These plant extracts were individually analyzed by our research group for their total polyphenolic contents, antioxidant activity, and cardioprotective potential. Present study was planned to evaluate their synergistic cardioprotective potential.

### 2.6. Animals

Male albino rabbits weighing 1–1.5 kg were selected for this study. Rabbits were kept under standard conditions of environment in the department of Clinical Medicine and Surgery (CMS), University of Agriculture, Faisalabad, Pakistan, and were allowed free access to standard diet and water. All international ethical considerations about animal studies were monitored during the experiment.

### 2.7. Experimental Protocol

Rabbits were kept for one week acclimatization period and then randomly divided into different groups. Each group comprised three rabbits.

*Group I (Normal Controls). *Rabbits were given standard diet only.

*Group II (Salbutamol Control Group).* Salbutamol was ingested to the rabbits (60 mg/Kg b.wt.) for two consecutive days to induce oxidative stress/myocardial cell necrosis.

*Group III (Baseline Group).* Herbal combination (100 mg/kg b.wt) was given orally to rabbits of this group once daily for three weeks.

*Group IV (Preventive Group). *Rabbits of this group were pretreated with plant combination 100 mg/kg b.wt. once daily for three weeks and then treated with two consecutive doses of salbutamol (60 mg/kg) orally. Blood samples were taken to evaluate any effect of herbal combination.

*Group V (Curative Groups). *Rabbits were treated with salbutamol (60 mg/kg) for two days to induce cardiotoxicity. Then these cardiointoxicated rabbits were treated with 200 mg/kg b.wt of plant combination once daily for five days and blood samples were collected daily to check the posttreatment effect of herbal mixture.

*Group VI (Standard Curative Group (Synthetic Drug)).* Rabbits were treated orally with salbutamol (60 mg/kg) for two days to induce cardiotoxicity. Then these cardiointoxicated rabbits were treated with a standard drug (Norvasc and Capoten) once daily for five days and blood samples were collected daily.

## 3. Biochemical Assessment

### 3.1. Estimation of Cardiac Biomarkers

Blood samples were taken from the jugular vein of rabbits and serum was separated for analysis of different cardiac biomarkers like lactate dehydrogenase (LDH), creatine kinase-MB fraction (CK-MB), aspartate transaminase (AST), and alanine transaminase (ALT). Among lipids total cholesterol, triglyceride, low density lipoprotein (LDL), and high density lipoprotein (HDL) were also estimated. All these analyses were performed with commercially available kits using chemistry analyzer (Semar S 1000-elite).

### 3.2. Estimation of Antioxidant Enzymes in Heart Tissues

After experimental period animals were slaughtered and heart tissues were separated and washed with isotonic saline. The tissues were homogenized in 10% ice cold phosphate buffer (pH = 7). Then this mixture was centrifuged and supernatant was collected for analysis of antioxidant enzymes like SOD, CAT, and GPx by following the method of Hameed et al. [19].

## 4. Toxicological Studies

### 4.1. Gross Pathology of Experimental Animal

Gross pathology of experimental animals was performed under the supervision of a veterinary doctor. Changes in weight and structure of heart, kidneys, liver, stomach, and lungs were noted.

### 4.2. Histopathological Analysis

Histopathological analysis was performed on the apical portion of the heart, lungs, kidney, and liver. Fresh tissues of these organs were excised and fixed in 10% formalin for 24 hours. Sections were cut into 5 *μ*m thickness and stained with hematoxylin and eosin. The sections were mounted and observed under light microscope with magnification of 200x for histological changes.

### 4.3. Statistical Analysis

The results were expressed as mean ± standard error of mean for three rabbits in each group. The statistical analysis was performed using Minitab 16.0. Analysis was made using one-way analysis of variance (ANOVA) followed by Tukey's comparison test. *P* value of <0.05 was considered statistically significant.

## 5. Results

### 5.1. HPLC Profile of Polyphenolic Contents

**Figure 1:**
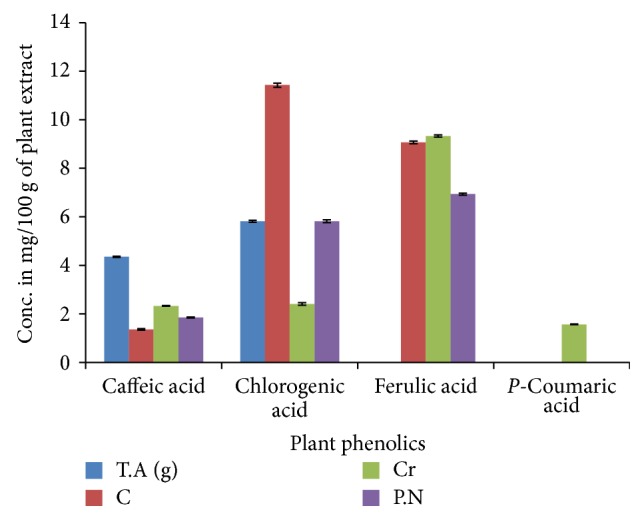
HPLC analysis of polyphenolic contents of four medicinal plants.

The amount of polyphenols identified in different medicinal plants has been shown in Figure 1.

Highest amount of caffeic acid was present in gemmo Arjun (4.352 mg/100 g of plant extract) followed by Crataegus (2.326 mg/100 g), Black Pepper (1.851 mg/100 g), and Cactus (1.361 mg/100 g).

Highest amount of Chlorogenic Acid was found in* Cactus grandiflorus* (Cactus) that was 11.429 mg/100 g of plant extract, while the concentration of Chlorogenic Acid was 9.118 mg/100 g in Black Pepper, 5.816 mg/100 g in gemmo Arjun, and 2.409 mg/100 g in Crataegus. Maximum amount of Ferulic acid was present in Crataegus (9.328 mg/100 g) followed by Cactus and Black Pepper in which the amount of Ferulic acid was 9.067 mg/100 g and 6.935 mg/100 g of plant extract, respectively.* P*-Coumaric acid acid was only present in Crataegus (1.568 mg/100 g) and was absent in all other plants.

### 5.2. Effect of Herbal Combination on Cardiac Markers (Enzyme) and Lipids

Cardioprotective potential of herbal combination was assessed through curative and preventive modes of treatment.

### 5.3. Preventive Cardioprotective Potential

In preventive mode of treatment herbal combination was fed orally for three weeks to experimental animals. After that, salbutamol was given (60 mg/kg b.wt.) for two consecutive days to induce oxidative stress which could untimely lead to cell necrosis, ventricular arrhythmia, and myocardial infarction that was confirmed by positive troponin test. Troponins are structural proteins of cardiac muscles which are secreted into blood with myocardial injury and are good markers for myocardial cell necrosis and myocardial infarction.

Salbutamol significantly (*p* < 0.05) increased the level of cardiac biomarker enzymes (CK-MB, AST, ALT, and LDH) in salbutamol induced control group as compared to animals of normal control. Increased level of these enzymes was due to the oxidative stress and myocardial cell necrosis caused by salbutamol. Prior administration of herbal mixture at the dose of 100 mg/kg significantly (*p* < 0.05) maintained the salbutamol induced elevated level of cardiac enzymes. A significant (*p* < 0.05) increase was observed in the levels of lipid profile (LDL, cholesterol, and triglycerides) in salbutamol induced control group as compared to normal control indicating hyperlipidemia, while level of HDL was decreased in salbutamol induced control group. Herbal combination prevented the increase of lipids in preventive group showing the lipid lowering effect of herbal supernatant. Herbal mixture also restored level of HDL, whereas rabbits of base line group showed nonsignificant changes in the level of cardiac biomarkers (Tables 2 and 3).

### 5.4. Curative Cardioprotective Potential

In curative mode of treatment, oxidative cardiotoxicity (myocardial cell necrosis) was induced in rabbits by giving orally two consecutive doses of salbutamol, which significantly (*p* < 0.05) increased the level of cardiac biomarkers (CK-MB, LDH, AST, and ALT) and lipids of experimental animals. This increased level was then subsequently decreased gradually by treating the animals with herbal mixture. After five days treatment animals were almost completely recovered, indicating the cardioprotective potential of herbal combination. The cardioprotective potential of herbal combination was comparable with synthetic standard drug. Five days treatment of cardio intoxicated rabbits with herbal combination also maintained salbutamol induced elevated level of lipids. Herbal combination restored the lipid level better than synthetic cardioprotective drug (Tables 4 and 5).

### 5.5. Effect of Herbal Mixture on Myocardial Antioxidants Enzymes

Results of antioxidant enzymes demonstrated that the level of all the three enzymes superoxide dismutase (SOD), catalase, and glutathione peroxidase was decreased significantly (*p* < 0.05) in salbutamol induced control group as compared to the animals of normal control group indicating high oxidative stress. Treatment of rabbits with herbal mixture restored the level of antioxidant enzymes. Polyphenolics rich herbal combination exhibited better potential in curative mode of treatment (Table 6).

## 6. Toxicological Studies

Toxicological study was performed through gross pathology and histopathological examination.

### 6.1. Gross Pathology

Results of gross pathology of various organs of different experimental groups of rabbits are given in Tables 7 and 8. These results demonstrated that the weight of different body organs of salbutamol induced control group was increased remarkably (*p* < 0.05) as compared to animals of normal control. The weight of body organs was normal in rabbits treated with herbal combination.

### 6.2. Histopathological Examination of Cardiac Tissues

**Figure 2:**
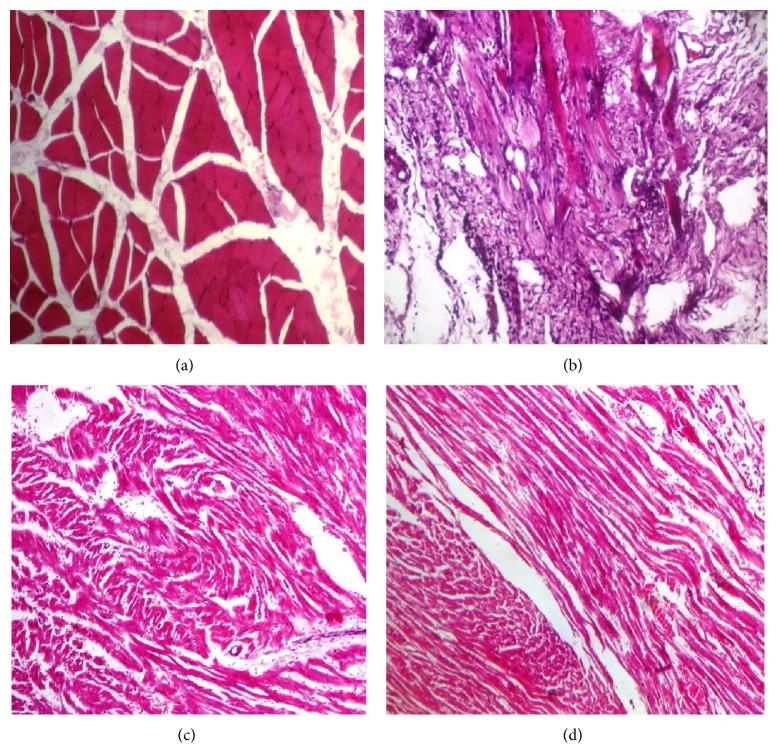
Histopathological architecture of heart of different experimental groups.

The histopathological architecture of heart from different experimental groups showed series of variations (Figure 2). In the normal control group, myocardial fibers were arranged regularly with clear striation. No apparent degeneration or necrosis was observed (Figure 2(a)). Histological section of salbutamol treated heart showed severe necrotic and degenerative changes and hyperchromatic and pyknotic nuclei as well as fibroblastic hyperplasia and thick connective tissue proliferation (Figure 2(b)). Heart tissues were normal in rabbits treated with herbal combination. Mild necrotic changes in cardiomyocytes were observed in curative mode of treatment (Figure 2(c)). An insignificant necrosis was examined in the heart of preventive group (Figure 2(d)). Rabbits of base line group also showed normal results.

## 7. Discussion

The present study revealed both imperative curative and preventive ways of cardioprotective potential. It explained the cardioprotective potential of herbal mixture of four plants in widely used catechol amine-induced model of myocardial cell necrosis in rabbits. In the present research a significant (*p* < 0.05) increase was observed in the level of cardiac enzymes (CK-MB, LDH, AST, and ALT) in salbutamol (catechol amine) induced control group as compared to animals of normal control group. Salbutamol, which has structural similarities with Isoproterenol (ISO), is a synthetic catecholamine and *β*-adrenergic receptor agonist. At high dose it has the ability to destruct myocardial cells and produce cardiotoxicity in experimental animals, as a result of disturbance in physiological balance between production of free radicals and antioxidant defense system [20]. Increases in the level of these enzymes were due to their leakage from the damaged heart tissues into the blood stream during myocardial necrosis because of myofibril degeneration and myocyte necrosis [21, 22]. It also caused cardiac dysfunction and increased lipid peroxidation along with an increase in the level of myocardial lipids and altered activities of the cardiac markers and antioxidant enzymes [23, 24].

Treatment of different groups of rabbits with herbal mixture significantly reduced the salbutamol-induced secretion of all cardiac diagnostic marker enzymes (CK-MB, LDH, AST, and ALT). This decreased level or reduction in the secretion of enzymes could be of enzymes could be due to repairing and maintenance of the myocardial cells membrane. Curative and preventive treatment of rabbits with polyphenolic enriched herbal combination significantly decreased the elevated cardiac enzyme. Polyphenols are potent antioxidant, neutralizing lipid free radicals and prevent decomposition of hydroperoxides into free radicals [25, 26]. Their cardioprotective potential may be due to scavenging of highly oxidized metabolites produced by salbutamol and stabilization of heart membrane by herbal combination with a consequent decrease in the leakage of these markers [21]. The tendency of these cardiac markers to become near the normal levels in prior and posttreated group is a clear manifestation of the cardioprotective potential of the herbal combination.

Significant (*p* < 0.05) elevated levels of total cholesterol, triglycerides, and low density lipoproteins (LDL) were observed in salbutamol induced control group indicating salbutamol induced hyperlipidemia. Highly oxidative metabolites of catecholamines lead lipid peroxidation which is the major destructive reaction in cellular mechanism of the myocardial ischemia. Highly oxidative metabolite of catecholamines like isoproterenol and salbutamol accelerates rate of peroxidation in membrane phospholipids and releases free fatty acids into plasma by the action of phospholipase A2 and it is a main causative aspect of salbutamol-induced hyperlipidemia [20]. The treatment of experimental animals with herbal mixture decreased salbutamol induced high level of lipids. With both ways of treatment the (preventive and curative) the levels of lipid profile reduced closer to the normal level because of the remedial action of herbal combination. The level of HDL was decreased in salbutamol control group indicating the reduction of good cholesterol, but in both curative and preventive group the HDL level increased significantly (*p* < 0.05) which is comparable with the normal control. It is hypothesized that HDL can eradicate cholesterol, from atheroma within arteries, and transfer it back to the liver for excretion or reutilization. That is why HDL-bound cholesterol is sometimes called “good cholesterol.” A high level of HDL-C protects against cardiovascular diseases, and low HDL cholesterol levels increase the risk of heart diseases [27]. Same trend of lipid profile was observed in many previous findings [16, 23, 28–31]. It is also obvious from the present findings that the prepared herbal combination gave overall better results as compared to the standard drugs because of its powerful antioxidant and nontoxic nature.

Level of antioxidant enzymes was significantly (*p* < 0.05) lower in salbutamol induced control group. Antioxidant enzymes are biomarker of oxidative stress. Production of highly reactive free radical species inhibited the activities of antioxidant enzymes [32]. Glutathione antioxidant system plays a fundamental role in cellular defense against reactive free radicals and other oxidant species. It protects the myocardial cellular membrane against oxidative damage by regulating the redox status of proteins in the cell surface membrane [4, 22]. In the present case decreased superoxide dismutase (SOD) activity in salbutamol control group may be due to excessive formation of superoxide anions or the decreased removal of superoxide anion, which can be harmful to the myocardium. The activities of H_2_O_2_ scavenging enzymes (CAT and peroxidase) also decreased significantly (*p* < 0.05) after the induction of salbutamol to the experimental rabbits. The activities of these enzymes can be explained by the fact that excessive superoxide anion may inactivate SOD, thus resulting in activation of H_2_O_2_ scavenging enzymes [4, 28]. Pretreatment of rabbits with herbal combination restored the level of endogenous antioxidant enzymes SOD, CAT, and peroxidase. Posttreatment of experimental animals with herbal mixture helped to regain the level of these enzymes near to normal. This can be correlated to the free radical scavenging potential of the herbal combination which protected the rabbits from reactive oxygen species. Several studies have reported the increase of endogenous antioxidants by herbal formulation or plants extracts in cardiovascular diseases [33, 34].

Gross/histopathological examination of different body organs such as heart, liver, lungs, and kidney proved the safe cardioprotective potential of herbal combination. Results of histopathological analysis are in line with many previous studies [35–39] and illustrated the cardioprotective potential and nontoxic nature of herbal combination.

## 8. Conclusion

The herbal combination prepared by mixing the appropriate ratio of four medicinal plants was administered to the rabbits suffering from salbutamol induced myocardial cell necrosis through both preventive and curative mode of treatments. All these four plants have been already evaluated individually, by our research group, for the cardioprotective potential. In the present study the green combination of the medicinal plants was made which showed better synergistic cardioprotective potential. Bioactive compounds present in different plants exert synergistic biofunctionalities in combination by interacting with one another, rather than acting alone. This herbal combination can be used as an alternative effective drug for the treatment of cardiovascular diseases because of its enriched polyphenolic contents and synergic cardioprotective potential.

## Figures and Tables

**Table 1:** Conditions used for HPLC analysis.

Column	Shim-Pack CLC-ODS (C-18), 25 cm × 4.6 mm, 5 *µ*m

Mobile phase	Gradient: A (H_2_O : AA—94 : 6, pH = 2.27), B (CAN 100%), 0–15 min = 15% B, 15–30 = 45% B, 30–45 = 100% B

Flow rate	1 mL/min

Detector	UV-visible detector. 280 nm

Temperature	RT

Range	Bipolar, 1250 mV, 10 samples per sec.

Detection	Gradient

**Table 2:** Preventive cardioprotective effect of herbal combination on cardiac enzymes in different experimental groups.

Groups	CK-MB (IU/L)	LDH (IU/L)	AST (IU/L)	ALT (IU/L)
Normal control	35.5 ± 0.32	545.8 ± 2.24	37.26 ± 0.37	45.6 ± 0.41
Salbutamol control group	80.4 ± 0.47^*∗*^	859.5 ± 3.57^*∗*^	113.5 ± 0.83^*∗*^	140.7 ± 0.63^*∗*^
Base line group	22.8 ± 0.27^#^	539.7 ± 4.01^#^	36.8 ± 0.54^#^	49.5 ± 0.84^#^
Herbal mixture + (salbutamol)	38.2 ± 0.48^#^	551.5 ± 2.07^#^	39.7 ± 0.55^#^	62.4 ± 1.05^#^

Results are expressed as Mean ± Standard Error of Mean (SEM) for *n* = 3.

^*∗*^Significantly different from normal control.

^#^Significantly different from salbutamol control.

**Table 3:** Preventive cardioprotective effect of herbal combination on lipid profile in different experimental groups.

Groups	Cholesterol (mg/dL)	Triglyceride (mg/dL)	LDL (mg/dL)	HDL (mg/dL)
Normal control group	42 ± 0.45	118.5 ± 1.43	26 ± 0.34	45.6 ± 0.47
Salbutamol control group	86.2 ± 0.39^*∗*^	342.4 ± 1.64^*∗*^	57.6 ± 0.63^*∗*^	32.4 ± 0.36^*∗*^
Base line group	49.5 ± 0.63^#^	164 ± 1.83^#^	19 ± 0.14^#^	55 ± 0.48^#^
Herbal mixture + salbutamol	55.5 ± 0.83^#^	203.8 ± 0.54^#^	29.5 ± 0.47^#^	43.7 ± 0.31^#^

Results are expressed as Mean ± Standard Error of Mean (SEM) for *n* = 3.

^*∗*^Significantly different from normal control.

^#^Significantly different from salbutamol control.

**Table 4:** Curative cardioprotective effect of herbal combination on cardiac marker (enzymes) in different experimental groups.

Enzyme	Day	Normal control	Salbutamol control	Salbutamol + herbal mixture	Standard drug
CK-MB (IU/L)	1	35.3 ± 0.50	80.3 ± 1.32^*∗*^	59.3 ± 0.49^#^	67.8 ± 1.06
	2	34.5 ± 0.35	81.5 ± 1.42^*∗*^	57.67 ± 0.54^#^	61.2 ± 1.67
	3	36.1 ± 0.54	83.7 ± 2.12^*∗*^	48.3 ± 0.76^#^	57.3 ± 2.32^#^
	4	32.7 ± 0.62	85.2 ± 1.37^*∗*^	39.25 ± 0.53^#^	49.8 ± 2.10^#^
	5	33.8 ± 0.47	82.8 ± 1.02^*∗*^	37.6 ± 0.67^#^	42.7 ± 1.84^#^

AST (IU/L)	1	37 ± 0.43	113.6 ± 0.86^*∗*^	95.3 ± 0.96	101.7 ± 2.5
	2	35.67 ± 0.70	114.1 ± 0.74^*∗*^	51.33 ± 1.76^#^	97.25 ± 2.47
	3	36.8 ± 0.23	113.7 ± 0.97^*∗*^	45.0 ± 1.65^#^	66.34 ± 3.10^#^
	4	35.1 ± 0.87	113.5 ± 0.75^*∗*^	41.67 ± 1.45^#^	61.9 ± 2.95^#^
	5	37.2 ± 0.56	114.3 ± 1.02^*∗*^	39.4 ± 2.01^#^	54.3 ± 1.95^#^

ALT (IU/L)	1	45 ± 1.43	142.4 ± 1.23^*∗*^	139 ± 1.87	147.3 ± 3.10
	2	43.3 ± 1.62	142.9 ± 1.54^*∗*^	136 ± 2.43	135.6 ± 2.73
	3	42.7 ± 1.45	143.7 ± 3.02^*∗*^	93.3 ± 2.56^#^	133.8 ± 2.74
	4	45.5 ± 1.56	141.8 ± 2.31^*∗*^	83.67 ± 2.12^#^	113 ± 2.43
	5	47.3 ± 1.76	144.2 ± 2.13^*∗*^	60.33 ± 1.98^#^	69.8 ± 3.45^#^

LDH (IU/L)	1	545.2 ± 2.43	859.2 ± 4.35^*∗*^	747.6 ± 4.71	810.5 ± 7.23
	2	549.5 ± 2.87	859.6 ± 3.84^*∗*^	609.7 ± 2.54^#^	771.5 ± 6.34
	3	542.8 ± 2.61	857.3 ± 4.71^*∗*^	588 ± 3.78^#^	634 ± 9.33
	4	547.2 ± 3.54	855.1 ± 3.42^*∗*^	567 ± 9.32^#^	588.5 ± 7.83^#^
	5	541.3 ± 2.69	860.3 ± 5.67^*∗*^	549.6 ± 5.43^#^	552.7 ± 5.99^#^

Results are expressed as Mean ± Standard Error of Mean (SEM) for *n* = 3.

^*∗*^Significantly different from normal control.

^#^Significantly different from salbutamol control.

**Table 5:** Curative cardioprotective effect of herbal combination on lipids in different experimental groups.

Enzyme	Day	Normal control	Salbutamol control	Salbutamol + herbal mixture	Standard drug
Cholesterol (mg/dL)	1	42.3 ± 0.73	102.0 ± 3.45^*∗*^	98.7 ± 1.33	104 ± 0.64
	2	42.7 ± 0.43	102.8 ± 3.87^*∗*^	86.3 ± 1.06^#^	101 ± 0.71
	3	45.3 ± 0.56	101.3 ± 2.56^*∗*^	80.0 ± 1.43^#^	76.5 ± 0.48^#^
	4	43.5 ± 0.37	100.8 ± 2.76^*∗*^	67.8 ± 1.01^#^	69.7 ± 0.82^#^
	5	44.25 ± 0.92	103.1 ± 1.99^*∗*^	53.4 ± 0.43^#^	56.4 ± 0.58^#^

Triglyceride (mg/dL)	1	118.7 ± 1.56	342.6 ± 3.07^*∗*^	326.7 ± 1.47	340.7 ± 1.19
	2	117.9 ± 2.62	341.8 ± 2.25^*∗*^	305.2 ± 1.94	338.8 ± 1.35
	3	118.1 ± 3.27	343.6 ± 2.52^*∗*^	273.8 ± 1.54^#^	321.4 ± 1.39
	4	119.1 ± 2.97	342.1 ± 2.87^*∗*^	236.5 ± 1.43^#^	212.3 ± 1.09^#^
	5	116.6 ± 3.11	340.2 ± 3.67^*∗*^	147.7 ± 1.65^#^	192.7 ± 1.62^#^

LDL (mg/dL)	1	26.1 ± 1.96	57.0 ± 0.38^*∗*^	51.67 ± 0.23	55.4 ± 1.26
	2	26.8 ± 1.62	56.8 ± 0.87^*∗*^	48.3 ± 0.27	49.4 ± 1.33
	3	23.6 ± 1.68	57.7 ± 0.59^*∗*^	47.7 ± 0.34	43.8 ± 1.93
	4	22.9 ± 0.99	55.9 ± 0.48^*∗*^	36.67 ± 0.41^#^	36.6 ± 1.35^#^
	5	24.1 ± 0.57	55.4 ± 0.79^*∗*^	25.33 ± 0.22^#^	35.8 ± 1.29^#^

HDL (mg/dL)	1	45.7 ± 1.66	31.5 ± 1.32^*∗*^	31.33 ± 0.43	33.3 ± 1.37
	2	43.9 ± 1.59	33.2 ± 1.61^*∗*^	33.5 ± 0.97	35.6 ± 1.40
	3	44.3 ± 1.39	32.4 ± 0.99^*∗*^	38.3 ± 0.68	38.33 ± 0.9
	4	42.8 ± 2.56	31.8 ± 2.01^*∗*^	42.1 ± 1.3	40.2 ± 0.86
	5	43.1 ± 1.84	32.4 ± 1.03^*∗*^	43.3 ± 1.04	41.5 ± 0.37

Results are expressed as Mean ± Standard Error of Mean (SEM) for *n* = 3.

^*∗*^Significantly different from normal control.

^#^Significantly different from salbutamol control.

**Table 6:** Level of antioxidant enzymes (Units/g of wt.) in different experimental groups of rabbit.

Antioxidant enzyme	Control	Salbutamol control	Herbal mixture + salbutamol (preventive)	Salbutamol + herbal mixture (curative)	Standard drug
Superoxide dismutase (SOD)	95.42 ± 0.54	49.73 ± 0.64^*∗*^	66.45 ± 0.69^#^	99.68 ± 0.86^#^	44 .54 ± 0.47
Catalase	403.07 ± 0.87	61.00 ± 0.58^*∗*^	62.00 ± 0.47	400.00 ± 1.74^#^	937.43 ± 1.46^#^
Peroxidase	810.3 ± 1.32	730 ± 1.04^*∗*^	1800 ± 1.76^#^	600 ± 1.26^#^	1205.7 ± 1.73^#^

Results are expressed as Mean ± Standard Error of Mean (SEM) for *n* = 3.

^*∗*^Significantly different from normal control.

^#^Significantly different from salbutamol control.

**Table 7:** Weight of different body organs of different experimental groups.

Groups	Heart	Liver	Lungs	Kidney	
				Right	Left
Normal control	2.5	20.6	4.7	5	5.1
Salbutamol control	5.1^*∗*^	34.2^*∗*^	11^*∗*^	7.2^*∗*^	8.1^*∗*^
Preventive group	2.5^#^	20.2^#^	5.1^#^	4.8^#^	4.9^#^
Curative group	3.3	33.8	7.5	5.2	4.4^#^
Standard drug	2.8^#^	41.1	9.1	5^#^	5.3

Results are expressed as Mean ± Standard Error of Mean (SEM) for *n* = 3.

^*∗*^Significantly different from normal control.

^#^Significantly different from salbutamol control.

**Table 8:** Gross pathology of different groups of experimental rabbits.

Groups	Heart	Liver	Lungs	Kidney	
				Right	Left
Normal control	Normal	Normal	Normal	Normal	Normal
Salbutamol control	Enlarged, hard, and necrosis	Normal	Congested	Slight necrosis congested	Hemorrhage and congested
Preventive	Normal	Normal	Normal	Normal	Normal
Curative	Slightly congested	Normal	Normal	Normal	Normal
Standard drug	Normal	Normal	Congested	Normal	Slight necrosis
